# Les mariages consanguins et leurs effets sur les maladies non transmissibles dans la population marocaine: étude transversal

**DOI:** 10.11604/pamj.2022.41.221.31273

**Published:** 2022-03-16

**Authors:** Khaddouj El Goundali, Chaimae Bouab, Loubna Rifqi, Milouda Chebabe, Abderraouf Hilali

**Affiliations:** 1 Hassan First University of Settat, Higher Institute of Health Sciences, Laboratory of Health Sciences and Technologies, 26000 Settat, Settat, Morocco

**Keywords:** Consanguinité, maladies non transmissibles, cancer, maladies cardiovasculaires, diabète, maladies respiratoires, insuffisance rénale chronique, Inbreeding, non-communicable diseases, cancer, cardiovascular diseases, diabetes, respiratory diseases, chronic renal failure

## Abstract

**Introduction:**

la consanguinité est reconnue dans de nombreuses études comme un facteur appréciable affectant la santé de l´individu sur plusieurs générations et pose un problème réel de santé publique. Cette étude vise à déterminer les effets de la consanguinité sur les maladies non transmissibles, en particulier la susceptibilité à une série de maladies chroniques et complexes dans la population marocaine.

**Méthodes:**

c´est une étude transversale analytique basée sur un sondage mené dans deux communes marocaines Bni hlal et Foum jemaa. L'échantillon étudié comportait 222 individus. Les rapports de côtes avec leurs intervalles de confiance à 95% ont été calculés pour la probabilité de maladie selon le statut de consanguinité. Le test du Chi 2 été utilisé pour vérifier l'association entre les variables catégorielles. Une valeur de P < 0,05 était considérée comme significative.

**Résultats:**

une fréquence de 43.2% des mariages consanguins a été enregistré chez les répondants contre 41% chez leurs parents. Nous avons remarqué que la consanguinité entre les parents augmente les chances de mariage consanguin parmi leurs progénitures (p = 0.01). La génération issue des parents consanguins a un risque plus élevé de maladies non transmissible telles que les cancers, le diabète, les maladies cardiovasculaires, les maladies respiratoires chroniques et l´insuffisance rénale chronique.

**Conclusion:**

la présente étude a révélé une augmentation de la prévalence des maladies non transmissibles dans la population consanguine. Cela peut confirmer le rôle des facteurs génétiques dans l'ensemble du spectre de la maladie et pas seulement pour les troubles mendéliens.

## Abstract (en)

**Introduction:**

inbreeding is recognized in many studies as an important factor impacting health across several generations. It poses a real public health problem. The purpose of this study is to determine the effects of inbreeding on non-communicable diseases, in particular susceptibility to a range of chronic and complex diseases in the Moroccan population.

**Methods:**

we conducted an analytical cross-sectional study based on a survey conducted in two Moroccan communes: BNI hlal and Foum jemaa. The sample studied consisted of 222 individuals. Odds ratios (ORs) with 95% confidence intervals were calculated for the probability of having the disease by the inbreeding status. The chi-square test was used to assess the relationship between the categorical variables. A p. value < 0.05 was considered significant.

**Results:**

a frequency of consanguineous marriages of 43.2% was recorded among respondents vs 41% among their parents. We found that inbreeding between parents increased the chances of consanguineous marriages among offsprings (p = 0.01). The generation of consanguineous parents had a higher risk of developing non-communicable diseases such as cancers, diabetes, cardiovascular diseases, chronic respiratory diseases and chronic renal failure.

**Conclusion:**

this study shows an increase in the prevalence of non-communicable diseases in the consanguineous population. This can confirm the influence of genetic factors upon the entire spectrum of the disease and not just inmendelian disorders.

## Introduction

Le choix d´un conjoint est considéré parmi les plus difficiles et les décisions les plus complexes que les individus sont amenés à effectuer un jour dans leur vie, il est porteur de stratégies qui mettent en jeu à la fois des intérêts personnels et collectifs [1]. Conséquemment, le choix du conjoint influe directement sur la répartition, la structure et l´hétérogénéité du patrimoine génétique de toute la population [2]. La consanguinité est en effet un cas particulier des liens matrimoniaux entre les conjoints. Selon Bittles [3, 4] un mariage est classé comme consanguin s´il a été contracté entre deux personnes biologiquement apparentées comme cousin au deuxième degré avec un coefficient de consanguinité F≤0,0156. Car l´influence génétique (le niveau d´homozygotie) dans l´union au-delà du cousin germain (F<0,0156) ne diffère que légèrement de celle observée dans la population générale.

Les unions consanguines sont adoptées depuis des siècles, elles constituent une fraction significative des mariages dans le monde entier [5]. Présentement, elles sont pratiquées par plus d´un milliard de la population mondiale avec des taux atteignant 20 à 50% [6] et même 80,6% dans certaines communautés [7]. Particulièrement dans les pays musulmans d´Afrique du Nord, de Moyen-Orient, d´Asie centrale et dans la plupart des régions de l´Asie du sud [5, 8] une ceinture transversale cheminant du Pakistan et de l'Afghanistan à l'Est au Maroc à l'Ouest, et en Inde du Sud [6]. Cette pratique est favorisée et respectée dans la plupart sinon dans toutes les communautés arabes [9]. En effet, C´est une tradition arabe et musulmane, au nom d´une sécurité financière et affective, mais au prix de la santé des enfants, qui courent le risque de maladies génétiques plus que d´autres [10]. Nombreux est variés sont les facteurs contribuant à la forte prévalence des mariages consanguins; à partir des résultats de plusieurs études empiriques, différents facteurs ont été identifiés comme déterminants important de la forte prévalence des mariages consanguins, à savoir: les facteurs socio-économiques, socioculturels, religieux, géographiques et démographiques [7].

L´union consanguine représente depuis longtemps un sujet controversé, avec une attention particulière portée aux effets néfastes sur la santé [11]. Particulièrement sur son impact sur la reproduction, la mortalité et la morbidité infantile et les maladies mendéliennes rares [7, 9], Néanmoins, des informations limitées sont disponibles sur le rôle possible de la consanguinité et des gènes récessifs dans les maladies non transmissibles courantes [6]. En dépit qu´aujourd´hui le plus gros problème de santé publique réside dans les maladies non transmissibles (MNT), elles représentent le mal du 21^e^ siècle et un défi pour tous les pays. Parmi 57 millions du total des décès mondiaux sont dus aux MNT, soit 71%, dont 15 millions sont des décès prématurés survenant entre 30 et 70 ans [12]. Elles progressent d´une manière exponentielle et menacent la croissance économique et le développement à l´échelle mondiale. Au Maroc, la situation est aussi alarmante qu´au niveau mondial. La transition épidémiologique et démographique se traduit par une augmentation de la charge de morbidité et de mortalité des maladies non transmissibles, en particulier les cancers, le diabète, les maladies cardiovasculaires, les maladies respiratoires chroniques et l´insuffisance rénale chronique [13]. Ces maladies sont la principale cause de mortalité avec 80% des décès toutes causes confondues, ce qui place le Maroc parmi les pays à forte mortalité dû aux MNT dans la région de la Méditerranée orientale [12]. La stratégie nationale multisectorielle de prévention et de contrôle des maladies non transmissibles 2019- 2029 vient dans un contexte mondial marqué par l´engagement des Etats membres des Nations Unies dans la prévention et la maîtrise des MNT en les définissant comme des priorités du développement durable [14]. Dans cette perspective, la présente étude vise à déterminer les effets de la consanguinité dans la population marocaine sur les maladies non transmissibles communes, en particulier la susceptibilité à une série de maladies chroniques et complexes, particulièrement les cancers, le diabète, les maladies cardiovasculaires, les maladies respiratoires chroniques et l´insuffisance rénale chronique.

## Méthodes

**Conception de l'étude:** il s´agit d'une étude transversale, descriptive et analytique basée sur un sondage menée dans deux communes marocaines Bni hlal et Foum jemaa, le milieu d´étude couvre la zone urbaine et la zone rurale. L´étude s´est étalée sur une période de 3 mois allant du mois de juillet au mois de septembre 2020.

**Population cible et plan d´échantillonnage:** la population cible est constituée par l´ensemble des individus non-célibataires âgés de 18 ans et plus.

**Critères d´inclusion:** les individus âgés de 18 ans et plus, qui avaient déjà été mariés et qui pouvaient être soit en union, veufs ou séparés au moment de l´enquête, de nationalité marocaine.

**Critères d´exclusion:** les individus âgés moins de 18 ans et qui n'ont jamais été mariés, qui parlent une autre langue que la langue locale et les individus ayant refusé de participer à l'étude. Un plan d´échantillonnage à deux degrés a été utilisé, dont les unités au premier degré constituées de deux établissements de Soins de Santé Primaires (ESSP) choisis aléatoirement dans chacune des communes sélectionnées. Au niveau des unités secondaires, seuls les sujets mariés âges de 18 ans et plus ont été éligibles. L´échantillonnage consiste à sélectionner au hasard tous les individus répondant aux critères d'inclusion qui se présentent au niveau des milieux d´étude.

**Calcul de la taille de l'échantillon:** un échantillon de 272 individus a été choisi sur la base de la formule suivante: n = z^2^ x p (1-p)/m^2^, n = taille de l´échantillon, z = niveau de confiance selon la loi normale centrée réduite (pour un niveau de confiance de 95%, z = 1,96), p = proportion estimée de la population qui présente la caractéristique, une estimation de 23,4% pour la prévalence de la consanguinité au Maroc telle que rapportée dans l´enquête Nationale [15], m = marge d´erreur tolérée (estimé à 5% pour la présente étude) [16]. Cette formule détermine le nombre de personnes n à interroger en fonction de la marge d´erreur m que l´on peut tolérer sur une proportion de réponses p. Sur les 272 personnes qui ont satisfait aux critères d'inclusion, 222 individus ont accepté de participer, soit un pourcentage de 81,6%, tandis que les autres sujets ont été exclus par ce que certains ont refusé de participer et d´autres ont soumis des questionnaires incomplets.

**Méthode de collecte des données:** vu le type et le but de cette étude, la méthode utilisée pour collecter les données est le questionnaire entrevu. Toutes les informations ont été collectées sur la base des entretiens structurés en face à face auprès des individus éligibles à l´étude. Le choix de cette méthode est justifié par la traduction des objectifs de l´étude en variables mesurables. Grace au questionnaire, la transcription écrite des réponses permet de les uniformiser et les classer ce qui assure une certaine fiabilité. C´est aussi une méthode qui renferme plus d´objectivité par l´élimination de l´influence de l´enquêteur. Le questionnaire a été conçu, validé par des experts au niveau du laboratoire. En outre, la validité du contenu et la validité apparente ont été testé.

**Les variables:** le questionnaire comprenait des questions relatives aux informations suivantes:

***Variables socio démographiques:*** âge, sexe, lieu de naissance, situation matrimoniale, résidence. Variables socioéconomiques; niveau d´instruction, profession du répondant, le revenu du ménage.

***Variables anthropologiques:*** lien de parenté du couple, et des parents, le degré de parenté des parents et du couple actuel.

***Variables sur l'état de santé:*** des questions ont été posées sur l´atteinte par les maladies non transmissibles notamment le cancer, le diabète, les maladies cardiovasculaires, les maladies respiratoires chroniques et l´insuffisance rénale chronique chez les répondants et leurs descendants.

**Contrôle des biais:** toutes les informations ont été recueillies sur la base d'entretiens structurés en face à face par des professionnels de santé qualifiés utilisant la langue locale. Afin de limiter un biais de mémorisation, nous avons utilisé plusieurs sources (ordonnance, carnet de santé), également nous avons éliminé les questionnaires incomplets. L'enquête a été menée un questionnaire conçu et validé par des experts au niveau du laboratoire et la validité du contenu et la validité apparente ont été testé.

**Méthode de traitement et d´analyse des données:** les données recueillies par le questionnaire sont traitées et analysées de façon rigoureuse. Les analyses statistiques sont effectuées à l'aide du logiciel Soft Package Social Sciences (SPSS). La relation entre les époux a été enregistrée et si leurs parents étaient consanguins. Les rapports de cotes avec leurs intervalles de confiance (IC) à 95% ont été calculés pour la probabilité de maladie selon le statut de consanguinité dans la génération actuelle ainsi que pour les descendants du répondant. Pour la génération actuelle, les cas ont été définis comme les enquêtés issus d'unions consanguines (la déclaration de la maladie se limite à la personne elle-même ou à ses frères et sœurs atteints de la maladie) et les témoins ont été définis comme les enquêtés issus d'unions non consanguines (la déclaration de la maladie se limite à la personne elle-même ou à ses frères et sœurs atteints de la maladie). Des définitions similaires ont été adoptés pour les descendants des répondants. Le test du chi carré a été utilisé pour vérifier l'association entre deux ou plusieurs variables catégorielles. Une valeur de p inférieure à 0.05 était considérée comme significative.

**Considérations éthiques:** le consentement libre et éclairé des participants à l´étude est obtenue après leur avoir expliqué que les données recueillies sont seulement exploitées dans un but de la recherche scientifique, le droit à l´anonymat et la confidentialité qui se traduit par l´engagement de garantir à tous les participants leurs anonymats tout au long de l´étude, le droit à l´autodétermination en reposant sur le fait d´informer et signaler à tous les participants à cette étude qu´ils ont le droit de décider librement et volontairement de leur adhésion ou non à l´étude, le traitement juste et objectif des données et, la neutralité et l´objectivité dans la présentation et la discussion des résultats.

## Résultats

Sur les 272 personnes qui ont satisfait aux critères d'inclusion, 222 individus ont accepté de participer, soit un pourcentage de 81,6%, tandis que les autres sujets ont été exclus par ce que certains ont refusé de participer et d´autres ont soumis des questionnaires incomplets. Sur les 222 individus éligibles interrogés dans cette étude, une fréquence de 43.2% des mariages consanguins a été enregistré chez la génération des couples actuelle contre 41% chez la génération des parents. Les caractéristiques générales de la population étudies sont décrites dans le Tableau 1. La majorité des sujets de l´étude avaient 45 ans et plus (38,7%), le sexe féminin représente 57,2%, plus de la moitié de la population soit 59% réside dans le rural, 25,2% sont des analphabètes et la moindre proportion (14,4%) avait un niveau des études supérieur. Environ un tiers de la population sans emplois. La répartition de la prévalence selon le niveau socio-économique du ménage montre le quintile le plus riche enregistre la prévalence la plus faible avec une proportion de 9% alors que le quintile le plus pauvre enregistre 22,5% et le second quintile enregistre la prévalence la plus élevée soit 27,5%.

**Tableau 1:** données sociodémographiques des sujets étudiés par consanguinité (N=222)

	Total N=222	Consanguins (N=91) (41 %)	Non consanguins (N=131) (59 %)	p value
	N (%)	N (%)	N (%)	
**Statut matrimonial**				
Marié	158 (71.2)	69 (31.1)	89 (40.1)	0.98
Divorcé	35 (15.8)	15 (6.8)	20 (9.0)	
Veuf	29 (13.1)	13 (5.9)	16 (7.2)	
**Age en année**				
Moins de 25 ans	46 (20.7)	18 (8.1)	28 (12.6)	0.8
26-34 ans	47 (21.2)	20 (9.0)	27 (12.2)	
35-44 ans	43 (19.4)	18 (8.1)	25 (11.3)	
45 ans et plus	86 (38.7)	41 (18.5)	45 (20.3)	
**Le sexe**				
Femme	127 (57.2)	55 (24.8)	72 (32.4)	0.89
Homme	95 (42.8)	42 (18.9)	53 (23.9)	
**Milieu de résidence**				
Urbain	91 (41)	37 (16.7)	54 (24.3)	0.45
Rural	131 (59)	60 (27.0)	71 (32.0)	
**Niveau d'instruction**				
Analphabète	56 (25.2)	26 (11.7)	30 (13.5)	0.99
Préscolaire	35 (15.8)	14 (6.3)	21 (9.5)	
Primaire	56 (25.2)	25 (11.3)	31 (14.0)	
Secondaire	43 (19.4)	18 (8.1)	25 (11.3)	
Supérieur	32 (14.4)	14 (6.3)	18 (8.1)	
**Statut professionnel**				
Salarié	42 (18.9)	17 (7.7)	25 (11.3)	0.53
Fonctionnaire	19 (8.6)	7 (3.2)	12 (5.4)	
Libérale	28 (12.6)	11 (5.0)	17 (7.7)	
Agriculture	15 (6.8)	10 (4.5)	5 (2.3)	
Autre	45 (20.3)	19 (8.6)	26 (11.7)	
Sans	73 (32.9)	33 (14.9)	40 (18.0)	
**Revenu du ménage**				
moins de 1930 DH	50 (22.6)	21 (9.5)	29 (13.1)	0.65
1930 à moins de 2892 DH	61 (27.5)	24 (10.8)	37 (16.7)	
2892 à moins de 4227 DH	60 (27.1)	29(13.1)	31 (14.0)	
4227 à moins de 6650 DH	31 (14)	16 (7.2)	15 (6.8%)	
6650 DH et plus	20 (9)	7 (3.1)	13 (5.9)	
**Consanguinité parentale**				
Oui	91 (41)	49 (22.1)	42 (18.9)	0.01
Non	131 (59)	48 (21.6)	83 (37.4)	

En termes de corrélas de consanguinité, l´inverse de l´association attendue avec la consanguinité a été trouvé pour les variables explicatives notamment l´âge, le sexe, le milieu de résidence, le niveau d´instruction, le statut professionnel et le revenu du ménage. Nous avons également remarqué que la consanguinité entre les parents augmente les chances de mariage consanguin parmi leurs progénitures (les couples étudiés) avec un p value de 0.01 Tableau 1.

Les données sur les tendances des niveaux de consanguinité dans la génération des répondants par rapport à la génération parentale sont présentées dans le Tableau 2. Le type de mariage consanguin le plus courant était le mariage entre cousins germains (21,2%) La deuxième catégorie la plus fréquente de mariages consanguins était les mariages entre cousins germains au premier degré (12,6%), tandis que 13,2% de tous les mariages ont eu lieu entre cousins plus éloignés (Tableau 2). Pour la génération des parents, 10,8% des mariages sont entre cousins germains, contre 9% et 5% pour les mariages entre cousins germains au premier degré et cousins germains au 2^e^ degré respectivement, alors que les mariages entre cousins plus éloignés représentaient 16,2%.

**Tableau 2:** consanguinité dans la génération actuelle par rapport à la génération parentale

Degré de consanguinité	La generation actuelle (N = 222 )	la génération parentale (N = 222 )
Non consanguin	126 (56.8%)	131 (59%)
Consanguin	96 (43.2%)	91 (41%)
Cousins germains	47 (21.2%)	24 (10.8%)
Cousins germains au 1^er^ degré	28 (12.6%)	20 (09 %)
Cousins germains au 2^e^ degré	8 (3.6%)	11 (05%)
Cousins plus éloignés	13 (5.9%)	36 (16.2)

La prévalence des maladies non transmissibles chez la génération actuelle et leur progéniture par accouplement consanguin et non consanguin est dévoilée dans le Tableau 3. Les données montrent que les répondants issus des parents consanguins présentaient un risque significativement plus élevé que les issus de parents non consanguins en ce qui concerne les maladies cardiovasculaires (OR=2,411 [IC à 95%: 1,392-4,177] p=0,002), le diabète (OR=1,954 [IC à 95%: 1,131-3,375] p=0,016), et le cancer OR=2,102 [IC à 95%: 1,084-4,077] p=0,026). Il y avait également une différence significative dans la prévalence entre la progéniture de parents consanguins et celle de parents non consanguins pour les cas de diabète (OR=2.889 [IC à 95%: 1.181-7.068] p=0.016), de cancer (OR=3.869 [IC à 95%: 1.192-12.558] p=0.017), et de l´Insuffisance rénale OR=3.140 [IC à 95%: 1.351-7.297] p=0.006). Toutes les maladies signalées étaient plus fréquentes chez les descendants de mariages consanguins.

**Tableau 3:** prévalence des maladies non transmissibles parmi la génération actuelle et la descendance par des unions consanguines et unions non consanguines

La generation actuelle	Consanguin N=91	Non consanguin N=131	RC (95%IC)	P value
Maladies cardiovasculaire	50	44	2,411 (1,392-4,177)	0,002
Diabete	46	45	1,954 (1,131-3,375)	0,016
Cancer	25	20	2,102 (1,084-4,077)	0,026
Asthme	42	47	1,532 (0,888-2,643)	0,124
Insuffisance rénale	30	29	1,730 (0,948-3,115)	0,072
**la progéniture**	**N=96**	**N=126**		
Maladies cardiovasculaire	11	13	1.102 (.471-2.580)	0.82
Diabete	16	8	2.889 (1.181-7.068)	0.016
Cancer	11	4	3.869 (1.192-12.558)	0.017
Asthme	32	28	1.705 (.939-3.097)	0.078
Insuffisance rénale	19	9	3.140 (1.351-7.297)	0.006

## Discussion

Le taux de consanguinité est relativement élevé dans la présente étude, avec un taux de 43,2% et les mariages entre cousins germains prédominent et représentent 21,2% de tous les mariages. Le taux de consanguinité dans la génération parentale était de 41%. Les données montrent qu'il existe une association significative entre la consanguinité chez les couples étudiés et le type de mariage de leurs parents (p = 0,01). Cette association entre les deux générations quant au choix matrimonial nous emmène à dire que le mariage consanguin reste un phénomène héritable des parents à leurs descendances. Cela montre que la tradition du mariage au sein de la famille est une pratique privilégiée et la nature de l'association dépend fortement du contexte culturel. Les véritables raisons invoquées pour justifier la préférence des mariages consanguins sont avant tout sociales. Dans les communautés à taux de consanguinité élevés, des études sociologiques indiquent que le mariage consanguin pourrait renforcer la stabilité des couples en raison d'une plus grande compatibilité entre le mari et la femme qui partagent les mêmes relations sociales après le mariage qu'avant le mariage, ainsi que la compatibilité entre le couple et l'autre famille [17].

Les prestataires de soins de santé et les généticiens ont généralement jugé l'impact global des mariages consanguins comme négatif lorsqu'il est évalué en termes de risques génétiques accrus pour la progéniture, par opposition aux avantages sociaux et économiques potentiels [18]. Ainsi, dans la littérature, un mariage consanguin, pouvant prédisposer la progéniture à des pathologies génétiques diverses, y compris les maladies non transmissibles courantes [6, 7, 19].

Les résultats de cette étude ont révélé une association entre consanguinité et les maladies non transmissibles telle que les maladies cardiovasculaires (OR=2,411 [IC à 95%: 1,392-4,177] p=0,002), le diabète (OR=1,954 [IC à 95%: 1,131-3,375] p=0,016), et le cancer OR=2,102 [IC à 95%: 1,084-4,077] p=0,026) Chez la génération des répondants et le diabète (OR=2.889 [IC à 95%: 1.181-7.068] p=0.016), le cancer (OR=3.869 [IC à 95%: 1.192-12.558] p=0.017), et l´insuffisance rénale OR=3.140 [IC à 95%: 1.351-7.297] p=0.006) chez la progéniture. Ce résultat est en accord avec les résultats de Bener *et al*. qui ont rapporté en 2107 qu'en raison de la consanguinité accrue, les familles des Arabes du Qatar ont joué un rôle majeur dans l'identification de nombreuses formes de maladies graves courantes chez l'adulte telles que le diabète 2,88 (1,73-4,79) <0,001, le cancer 5.18 (2.62-10.25) <0,001, maladies cardiaques 2,89 (1,73-4,79) 0,001, asthme 4,54 (2,45-8,41) <0,001, hypertension 3,55 (1,96-6,45) <0,001. En outre, au Bangladesh, des taux plus élevés de plusieurs maladies multifactorielles dans le groupe consanguin ont été observés, notamment l'asthme bronchique, les troubles rénaux. Un total de 84 enfants, dont 57 du groupe consanguin, présentaient des comorbidités [20]. Des études à long terme menées sur les îles de l'Adriatique, en Croatie, ont indiqué une association positive entre la consanguinité et un très large éventail de troubles courants, particulièrement l'hypertension, les maladies coronariennes, les accidents vasculaires cérébraux, le cancer et l'asthme [21].

En Inde, Bhasin et Kapoor ont montré que Le risque de maladies cardio-métaboliques est plus élevé chez les descendants de couples consanguins (Près de trois fois), et il y a une augmentation significative de la prévalence des maladies courantes de l'adulte, spécialement les maladies cardiaques OR=2.65 [IC: 1.02-6,85] p=0,044, le diabète OR=2.44 [IC: 1.26-4,76] p=0,009 et l'hypertension [OR=2.62: 1.39-4,94] p=0,003) [22]. À l'opposé de ces études, une étude sur la population arabe d'Israël n'a montré aucun effet de la consanguinité sur les taux d'affections courantes, notamment le diabète sucré, infarctus du myocarde, asthme bronchique [23]. Ce résultat est supporté par des autres études qui n'ont pas trouvé de corrélation notable entre la consanguinité et les troubles multifactoriels [24, 25]. De même, Bener *et al*. ont montré que la consanguinité dans la population arabe étudiée n'a pas d'impact sur le développement du cancer [26]. Dans le même sens, aux Émirats arabes unis, la consanguinité était associée à une réduction du risque global de cancer [27].

Dans la littérature L'impact de la consanguinité sur les troubles multifactoriels chez l'homme est largement inconnu et fait débat, et surtout que les maladies non transmissibles courantes telles que les maladies cardiovasculaires, le cancer, le diabète, l´asthme sont étiologiquement hétérogènes, avec une transmission multifactorielle suspectée dans la plupart des familles et des cas. Les gènes à haute susceptibilité pourraient jouer un rôle important dans l'expression d'une maladie complexe, et si ces gènes sont rares et transmis de manière autosomique récessive, la consanguinité pourrait être un facteur déterminant [6, 28]. Certains chercheurs prédisent que la consanguinité chez l'homme pourrait influencer une large catégorie de troubles complexes, davantage si la composante génétique de la maladie est principalement due à un grand nombre de variantes rares dans de nombreux gènes selon l'hypothèse maladie commune/variante rare [21, 28]. En effet, comme la plupart des variantes génétiques identifiées provoquant des maladies complexes chez l'homme sont partiellement récessifs [29], il semble que la consanguinité pourrait augmenter le risque de maladie en augmentant l'homozygotie à de divers loci génétiques avec de petits effets délétères sur les voies homéostatiques [21, 24].

La corrélation entre la consanguinité et les maladies non transmissibles reste peu concluante et même contradictoire. Des études plus approfondies sont nécessaires pour confirmer ou infirmer ces résultats. L´association de la consanguinité avec les troubles complexes peut être étudiée en utilisant différentes approches. Par exemple, les enquêtes épidémiologiques pourraient comparer la fréquence d'un trouble dans la descendance de parents cousins germains avec celle de parents non apparentés et le renforcement par des études plus approfondies qui permettront l´identification des racines génétiques spécifiques de la maladie à l'aide de techniques moléculaires qui conduit au développement rapide de tests prédictifs qui peuvent dire aux parents si leur futur enfant a hérité de gènes défectueux qui le prédisposeront à une maladie spécifique plus tard dans la vie [30].

Les résultats présentés dans ce rapport devraient servir de plateforme de référence pour des études ultérieures visant à comprendre le rôle des mariages consanguins dans le comportement reproductif et les maladies héréditaires, multifactorielles et congénitales. Cependant, il existe plusieurs limites. Premièrement, Parmi les limites de la présente étude, mentionnons le recours à un devis transversal, qui empêche de tirer toute conclusion sur les relations causales. Aussi, l'échantillon de l'étude est basé sur les visites des ESSP. Troisièmement, la majorité de l'échantillon de l'étude était constituée de femmes; par conséquent, les résultats peuvent ne pas être généralisables à la population de tous les sujets mariés. En outre, il convient de noter qu'il pourrait y avoir un certain biais dans les données associées à la déclaration de maladies qui peut survenir plus tard dans la vie.

## Conclusion

La présente étude a révélé une incidence plus élevée de certaines maladies dans la population consanguine avec une augmentation de la prévalence des maladies non transmissibles telles que les cancers, le diabète, les maladies cardiovasculaires, les maladies respiratoires chroniques et l´insuffisance rénale chronique. Nos données suggèrent que l'incidence accrue des troubles communs et complexes est un facteur supplémentaire à considérer comme un inconvénient du mariage consanguin, en plus des risques accrus de troubles mendéliens récessifs, d´où la nécessité de mettre en place un programme d´éducation sanitaire de masse et de conseil génétique dans le pays.

### Etat des connaissances sur le sujet

- Les maladies non transmissibles sont la principale cause de mortalité au niveau mondiale, elles sont également responsables de 60% de la charge mondiale de morbidité surpassant les autres problèmes de santé du 21^e^siècle;
- Jusqu'à présent, peu d'études ont été publiées sur les effets de la consanguinité sur maladies non transmissibles courantes qui représentent la plus grande partie du fardeau de la santé publique mondiale;
- Dans la littérature, il n'y a pas d'accord sur l'effet de la consanguinité sur les maladies non transmissibles courantes.

### Contribution de notre étude à la connaissance

- À notre connaissance, la présente étude est la première à décrire l´effet de la consanguinité sur les maladies non transmissibles dans la population marocaine;
- Les résultats présentés dans ce rapport devraient servir de plateforme de référence pour des études ultérieures visant à comprendre le rôle des mariages consanguins dans le comportement reproductif et les maladies héréditaires, multifactorielles et congénitales.

## Remerciements

Nous tenons à remercier sincèrement tous les participants à l'étude, leurs contributions favorables ont facilité l'atteinte des objectifs de travail malgré la situation pandémique. Nous remercions les décideurs, les médecins généralistes et les infirmières des ESSP pour leur compréhension et leur contribution à faciliter l'accès et la collecte de données.
